# Leg muscle strength is reduced and is associated with physical quality of life in Antineutrophil cytoplasmic antibody-associated vasculitis

**DOI:** 10.1371/journal.pone.0211895

**Published:** 2019-02-04

**Authors:** Arno C. Hessels, Johannes H. van der Hoeven, Jan Stephan F. Sanders, Elisabeth Brouwer, Abraham Rutgers, Coen A. Stegeman

**Affiliations:** 1 Department of Internal Medicine, Division of Nephrology, University of Groningen, University Medical Center Groningen, Groningen, The Netherlands; 2 Department of Neurology, University of Groningen, University Medical Center Groningen, Groningen, The Netherlands; 3 Department of Rheumatology and Clinical Immunology, University of Groningen, University Medical Center Groningen, Groningen, The Netherlands; University of the West of England Bristol, UNITED KINGDOM

## Abstract

**Objective:**

Physical quality of life is reduced in ANCA-associated vasculitis (AAV). This study aims to investigate whether this may be explained by reduced muscle strength and physical activity resulting from disease damage and steroid myopathy.

**Methods:**

Forty-eight AAV patients were sequentially included from the outpatient clinic. Patients in different stages of disease and treatment underwent measurements of muscle strength and anthropometric parameters. Patients filled in physical activity (Baecke) and quality of life questionnaires (RAND-36) and carried an accelerometer for a week. Muscle strength and physical activity were compared to quality of life, prednisolone use and disease duration.

**Results:**

Most AAV patients had lower knee extension (76%) and elbow flexion (67%) forces than expected based on healthy norms. Also, physical (P<0.001) and mental (P = 0.01) quality of life were significantly reduced compared to healthy norm values. Lower knee extension force (P = 0.009), younger age <70 (P<0.001) and relapse of vasculitis (P = 0.003) were associated with lower age-adjusted physical quality of life. Lower Baecke index (P = 0.006), higher prednisolone dose (P = 0.005) and ENT involvement (P = 0.006) were associated with lower age-adjusted mental quality of life. Leg muscle strength showed no association with current or cumulative prednisolone use. Disease duration was longer in patients with knee extension force below healthy norms (P = 0.006).

**Conclusion:**

Knee extension force and physical activity are positively associated with quality of life in AAV. Knee extension force decreases with longer disease duration, suggesting that disease- and treatment-related damage have a cumulative negative effect on muscle strength.

## Introduction

ANCA-associated vasculitis (AAV) is a group of primary vasculitides associated with inflammation of the small and medium sized blood vessels. The most frequent forms are Granulomatosis with Polyangiitis (GPA, formerly Wegener’s Granulomatosis) and Microscopic Polyangiitis (MPA) [1].

Mortality has drastically decreased after introduction of immunosuppressive therapy and most patients can now be brought into remission. Unfortunately, the disease and its treatment are associated with damage that accumulates with prolonged disease duration, recurrent disease episodes and treatment exposure [2,3].

Quality of Life (QoL), especially physical QoL, is reduced in AAV patients compared to the general population [4–6]. It is important to identify modifiable factors associated with health-related QoL [5,7], as these factors will guide development of new treatments that improve the outcome.

One of the factors previously associated with reduced physical QoL is prednisolone use [4,5]. A well-known adverse effect of glucocorticoids (GCs) such as prednisolone is skeletal muscle atrophy [8]. GC-induced skeletal muscle atrophy results from a combination of reduced protein synthesis and increased muscle proteolysis [8,9]. Mainly fast-twitch (type II) muscle fibres are affected [8]. Proximal muscles are more severely affected than distal and cranial muscles [9]. GC-induced skeletal muscle atrophy develops after approximately 4 weeks of therapy, and is most frequently seen with higher doses of GCs (prednisolone 40-60mg/d or equivalent doses of other GC) [9].

GC-induced skeletal muscle atrophy might partly explain the relation between prednisolone use and impaired physical QoL in AAV. In our clinical experience, many patients with AAV suffer from a significant loss of leg muscle strength during prednisolone treatment. They report difficulties rising from a chair and walking stairs. In several studies that focused on patient perspectives, patients reported muscle weakness as an important disease burden [10,11]. In a study by Newall et al, AAV patients had a reduced exercise capacity, which correlated with quadriceps force [12]. These findings suggest an impact of leg muscle force on exercise performance in AAV patients, which might in turn affect QoL.

Muscle strength can be improved through exercise [13]. Therefore, reduced muscle strength and an association of muscle strength with QoL in AAV patients would warrant intervention studies regarding exercise programs or improvement of muscle strength for this population.

The first goal of this cross-sectional study performed at the outpatient clinic of the Vasculitis Expertise Center Groningen was to investigate the relation between leg muscle force, physical activity and health-related QoL in AAV patients. The second goal was to study the relation of leg muscle force with disease duration and treatment exposure, especially GC treatment.

## Patients and methods

### Study population

GPA and MPA patients were recruited from the outpatient clinic of the University Medical Center Groningen (UMCG) between July 2015 and October 2017. Patients were eligible for inclusion if they met the following inclusion criteria: age ≥18 years, diagnosis of Granulomatosis with Polyangiitis (GPA) or Microscopic Polyangiitis (MPA) according to the Chapel Hill criteria [1], first diagnosis or most recent relapse of disease activity within 3 years prior to inclusion, and treatment with a high dose of prednisolone (1mg/kg/day, tapered according to local protocol) and an additional immunosuppressive drug (e.g., cyclophosphamide, rituximab) as induction therapy. Patients with comorbidity causing reduced mobility or muscle strength and patients with active disease were excluded. More specifically, patients with neurological disease (cerebrovascular accident, hernia nuclei pulposi, peripheral motor nerve damage, critical illness neuropathy), pulmonary disease (dyspnea and activity limitations due to pulmonary involvement of AAV, chronic obstructive pulmonary disease, restrictive pulmonary disease), cachexia and fractures (vertebral fractures, radial fracture) were excluded. All patients signed written informed consent for participation in the study. The study was performed according to the principles of the Declaration of Helsinki and has received ethical approval by the local Medical Ethical Committee of the University Medical Center Groningen (METc 2015/184).

### Design

All patients received an initial cross-sectional measurement after a regular visit to the outpatient clinic. Patients with a prednisolone dose ≥30mg/d at the first study visit were approached and asked informed consent for a follow-up measurement at a later time point, at a low prednisolone dose (< = 2.5mg/d), or after discontinuation of prednisolone.

### Measurements

#### Demographic and clinical characteristics

Demographic data and clinical characteristics were collected from the patients’ medical records. The following data were collected: age, sex, diagnosis (GPA or MPA), ANCA specificity (PR3, MPO, negative), disease activity at most recent disease episode as measured by the Birmingham Vasculitis Activity Score (BVAS) version 3 [14,15], ear-nose-throat (ENT) and neurological vasculitis activity (derived from BVAS), drug used for induction therapy, drug used for maintenance therapy, prednisolone use (initial dose (mg/d), current dose (mg/d), cumulative dose of last six months (g)), C-reactive protein (CRP) levels (mg/l) and 24-hour urinary creatinine excretion (mmol/24h).

#### Measurement of muscle strength

Isometric knee extension, elbow flexion and hip flexion strength were measured using a handheld dynamometer (CIT Technics, Groningen, The Netherlands) and expressed in Newton (N), as described by Van der Ploeg et al [16]. Test positions for the muscle strength measurements are the same as described by Bohannon [17] and shown by Douma et al [18]. The ‘break’ method was used, in which the researcher slightly overcomes the maximum force of the subject [19]. The measurements were performed by four operators. All operators received instructions and training before performing measurements for the study. Hand-held dynamometry of hip and knee force has previously been shown to have a good to excellent (ICC > = 0.75) intra- and inter-rater reliability as well as a good to excellent concurrent validity compared to gold-standard measurement using an isokinetic dynamometer [20]. The measurements were performed twice on each side, alternating measurements between sides. The highest measured value per muscle group was used as the maximum muscle force of that muscle group. Expected elbow flexion and knee extension forces for each patient were calculated based on age, sex, height and weight using a regression equation derived from a Dutch healthy population sample [18]. Elbow flexion and knee extension force were expressed as a percentage of this expected value.

#### Bioelectric impedance analysis

Bioelectric Impedance Analysis (BIA) was performed using electrodes on the right hand and foot using the Bodystat Quadscan 4000 (Bodystat Ltd, British Isles). The fat free mass index (kg/m^2) was calculated using a built-in formula of the Bodystat.

#### Physical activity

The Baecke questionnaire was used as generic (*i*.*e*., not disease-specific) self-report measure of physical activity. It has been validated for use in the general Dutch population [21,22]. The Baecke questionnaire measures physical activity at work, leisure time and sports. The scores are summarised into work, sports and leisure time indices which are added into a total score. Calculations have been described by Baecke et al [21]. The intensity levels of sports were derived from the Ainsworth compendium [23,24].

The Actiwatch 7 (Camntech, Papworth Everard, United Kingdom) was used as an objective measure of physical activity. Accelerometry is a reliable method for measurement of physical activity in patients with rheumatic disease [25]. Participants were instructed to wear the accelerometer day and night on the non-dominant wrist, except for activities involving water, such as swimming or taking a bath. The accelerometer output was expressed as the average kilo-counts per waking day.

#### Health-related quality of life

Health-related quality of life (HRQoL) was assessed using the RAND-36 questionnaire. This questionnaire contains eight subscales with scores ranging from 0 to 100. These can be further summarised into a physical component summary (PCS) and mental component summary score (MCS), which are calculated in such a way that the reference population has a mean of 50 and a standard deviation of 10 [26]. Age-adjusted reference values used for calculating the PCS and MCS were derived from a Dutch general population sample [27]. Background information on the questionnaire and its subscales can be found elsewhere [28].

### Statistics

All analyses were performed using SPSS Statistics version 23 (IBM). A two-sided P-value <0.05 was considered statistically significant. Variables were presented as n (percent [%]) or median (interquartile range [IQR]). Reliability of muscle strength measurements was assessed by comparing repeated muscle strength measurements using intraclass correlation coefficients (ICC) with an absolute agreement definition. Point estimates of ICC were assessed as poor (<0.50), moderate (0.50–0.74), good (0.75–0.89) or excellent (> = 0.90) as described previously [20]. Muscle strength and physical activity were compared with all subscales of the RAND-36, prednisolone dose and disease duration using Spearman Rank correlation. Disease duration was compared between patients with muscle strength below and above 100% of their predicted value[18]. For patients who received paired measurements, muscle strength (N) and self-reported physical activity were compared between the first (prednisolone dose >30mg/d) and second (prednisolone dose < = 2.5mg/d) visits using the Wilcoxon signed rank test. Finally, linear regression was performed with the age-adjusted PCS and MCS of the RAND-36 as dependent variables and previously reported factors associated with these scores in AAV [5,7], as well as muscle strength and physical activity, as independent variables in univariable analysis. Subsequently, a multivariable model was built for age-adjusted PCS and MCS with statistically significant factors from univariable analysis, as well as previously reported potential confounders [5,7], included as independent variables in the model. For age-adjusted PCS, these potential confounders were: age >69 years, sex, current prednisolone dose >5mg/d, nervous system involvement and previous vasculitis relapse. For age-adjusted MCS, potential confounders were: age >69 years, sex, current prednisolone dose >5mg/d, ENT involvement and previous vasculitis relapse.

## Results

**Fig 1:**
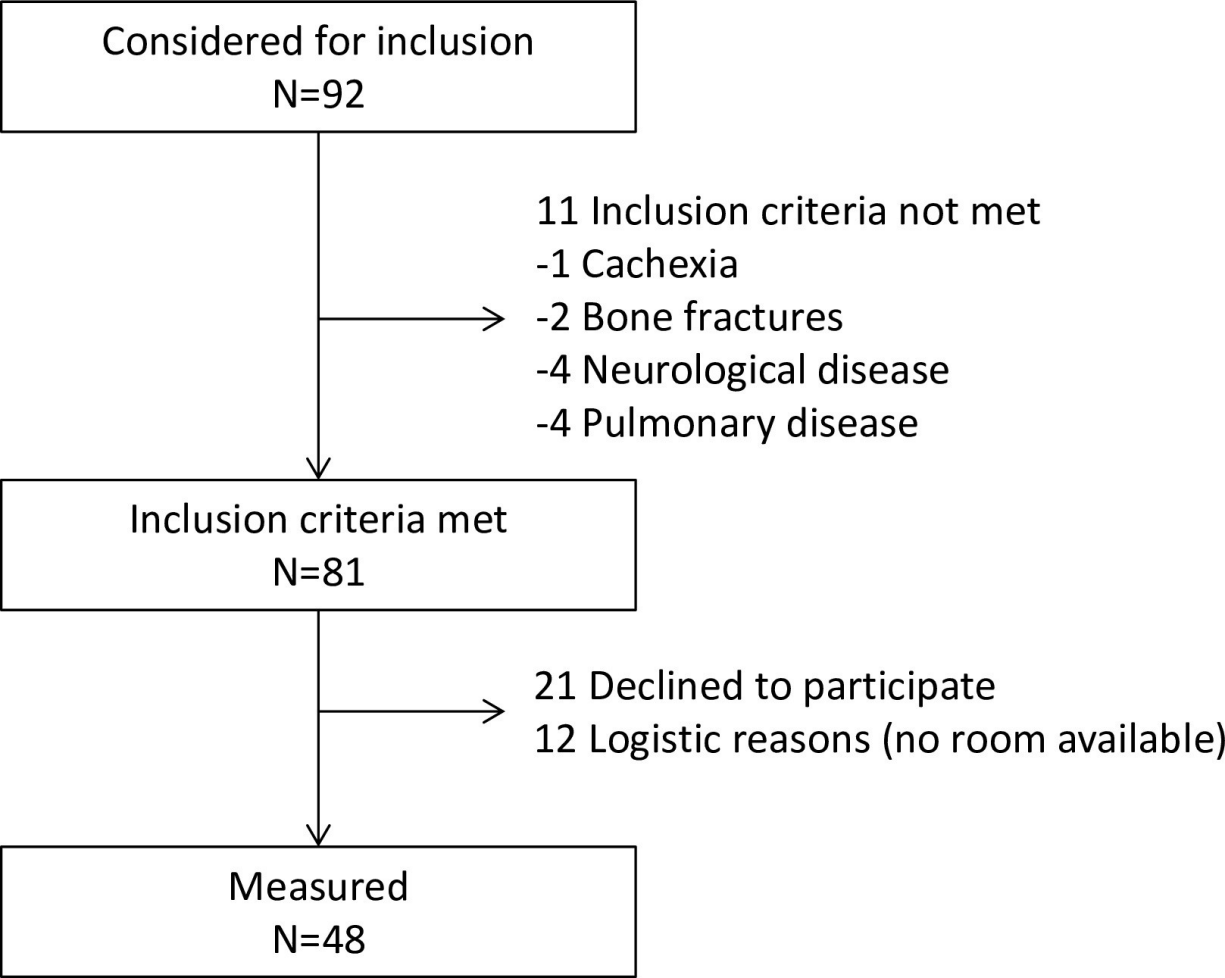
Flowchart of patient selection.

In total, 92 patients were considered for inclusion. A flowchart of inclusion is shown in **Fig 1**. Characteristics of the 48 patients included in the study are shown in **Table 1**. At the day of measurement, 12 patients (25%) had an elevated CRP (≥5mg/l). Incomplete variables include fat free mass index (present for 37/48, 77%), urinary creatinine excretion (present for 43/48, 90%) and total accelerometer score (present for 28/48, 58%).

**Table 1:** Patient characteristics.

	N (%) or Median (IQR)			
Variable	All	New	Relapsing	P-value
	(n = 48)	(n = 13)	(n = 35)	
Age (years)	62 (52–69)	64 (56–69)	61 (51–71)	0.86
Sex (n, % male)	25 (52%)	5 (39%)	20 (57%)	0.34
Diagnosis (N, % GPA)	41 (85%)	9 (69%)	32 (91%)	0.08
ANCA specificity (n,%)				0.30
Proteinase 3 (PR3)	35 (73%)	8 (62%)	27 (77%)	
Myeloperoxidase (MPO)	11 (23%)	5 (39%)	6 (17%)	
Negative	2 (4%)	0 (0%)	2 (6%)	
Treatment duration (weeks)	35 (11–104)	30 (9–62)	36 (14–119)	0.31
Months since diagnosis	83 (17–194)	7 (2–14)	141 (76–203)	<0.001***
Induction therapy (n, %)				0.001**
Cyclophosphamide	21 (44%)	11 (84%)	10 (29%)	
Rituximab	14 (29%)	0 (0%)	14 (40%)	
Other	13 (27%)	2 (16%)	11 (31%)	
Prednisolone	48 (100%)	13 (100%)	35 (100%)	
Currently on prednisolone	33 (69%)	6 (46%)	27 (77%)	0.08
Prednisolone dose (mg/d)	10.0 (0.0–28.8)	0.0 (0.0–50.0)	10.0 (3.8–25.0)	0.72
Cumulative prednisolone (g in last six months)	1.8 (0.1–3.3)	1.1 (0.0–3.8)	1.9 (0.6–2.9)	0.71
BVAS (diagnosis/relapse)	13 (11–17)	17 (13–21)	12 (8–16)	0.01*
CRP (mg/l) (at day of visit)	2.0 (0.7–5.2)	1.8 (0.8–2.8)	2.2 (0.6–5.5)	0.75

*P<0.05,

**P<0.01,

***P<0.001.

In total, seven patients have received paired measurements. Median prednisolone dose of these patients was 40mg/d (IQR 30–60) at the first measurement, versus 0mg (IQR 0–2.5) at follow-up. Median time between measurements was 6 months (IQR 4–25).

### Muscle strength and quality of life

Out of all 48 patients, 46 had a successful measurement of knee extension. Overall intra-rater reliability was good to excellent. Intraclass correlations, overall and per assessor, are shown in **S1 Table**. Of 46 measured patients, 35 (76%) had a knee extension force less than 100% of their predicted value based on age, sex, height and weight. Elbow flexion force was below 100% of predicted for 30/45 measured patients (67%).

Fat free mass index (kg/m^2) showed a significant positive correlation with hip flexion (Rho = 0.56, P<0.001), knee extension (Rho = 0.51, P = 0.001) and elbow flexion force in N (Rho = 0.68, P<0.001). Urinary creatinine excretion (mmol/24h) also showed an association with hip flexion (Rho = 0.47, P = 0.002), knee extension (Rho = 0.47, P = 0.002) and elbow flexion force (Rho = 0.33, P = 0.04).

Knee extension force (% of predicted) showed a significant positive correlation with the RAND-36 subscales physical functioning (Rho = 0.31, P = 0.04), physical role functioning (Rho = 0.34, P = 0.02), emotional role functioning (Rho = 0.31, P = 0.04) and general health (Rho = 0.30, P = 0.04). Elbow flexion force (% of predicted) did not show a significant correlation with any subscale of the RAND-36.

### Physical activity and quality of life

Baecke score showed a positive trend with hip flexion force (Rho = 0.29, P = 0.05), but not with elbow flexion (Rho = 0.22, P = 0.15) or knee extension force (Rho = 0.20, P = 0.18). Accelerometer counts did not show a correlation with muscle strength (not shown).

Baecke total score showed positive associations with the RAND-36 subscales physical functioning (Rho = 0.32, P = 0.04), role limitations (physical problem) (Rho = 0.33, P = 0.03), role limitations (emotional problem) (Rho = 0.46, P = 0.001), mental health (Rho = 0.41, P = 0.005) and general health (Rho = 0.42, P = 0.003), as well as positive trends with vitality (Rho = 0.28, P = 0.06) and pain (Rho = 0.26, P = 0.08). Accelerometer counts did not show a significant correlation with any subscale of the RAND-36.

### Associations of health-related quality of life

The age-adjusted PCS of the RAND-36 was significantly lower in AAV patients (mean 42, SD 10) compared to Dutch norm values (mean 50, SD 10, P<0.001). To a lesser degree, this was also true for the age-adjusted MCS (mean 46, SD 9, versus mean 50, SD 10; P = 0.01). In univariable linear regression, knee extension force, Baecke total score and neurological vasculitis activity at most recent disease episode (peripheral neuropathy (n = 4) or cranial nerve palsy (n = 1)) were associated with the age-adjusted PCS. Prednisolone dose >5mg/d, ENT vasculitis activity and lower Baecke score were associated with lower age-adjusted MCS, see **Table 2**.

**Table 2:** Univariable linear regression for quality of life in ANCA associated vasculitis.

Independent variables	Physical component summary			Mental component summary		
	B (95% CI)	P	Adj. R^2^	B (95% CI)	P	Adj. R^2^
Relapsing (versus new)	-6 (-13 to 0)	0.07	0.05	-1 (-7 to 6)	0.87	-0.02
Prednisolone dose >5 mg/d	-3 (-9 to 3)	0.35	0.00	-7 (-12 to -2)	0.01*	0.11
CRP ≥5mg/l	-4 (-11 to 3)	0.23	0.01	3 (-3 to 9)	0.25	0.01
ENT vasculitis activity	-1 (-7 to 6)	0.87	-0.02	-5 (-10 to 0)	0.04*	0.07
Neurological vasculitis activity	-12 (-22 to -1)	0.03*	0.08	-3 (-13 to 6)	0.49	-0.01
Knee extension (% of predicted)	0.1 (0.0 to 0.3)	0.04*	0.07	0.1 (0.0 to 0.2)	0.17	0.02
Elbow flexion (% of predicted)	0.1 (0.0 to 0.2)	0.06	0.06	0.0 (-0.1 to 0.1)	0.46	-0.01
Baecke total	3 (1 to 5)	0.01*	0.12	3 (1 to 4)	0.008**	0.13

Univariable linear regression of factors potentially associated with age-adjusted physical and mental quality of life. B coefficient of independent variable in linear regression. Adj. R^2^ adjusted R squared.

*P<0.05.

**P<0.01.

In multivariable linear regression, age < = 69 years, previous relapse of vasculitis, elevated CRP and lower knee extension force were associated with lower age-adjusted physical quality of life, with a total adjusted R^2^ of 0.55 for the regression model. Prednisolone dose >5mg/d, ENT involvement and lower Baecke total index were associated with lower age-adjusted mental quality of life, with a total adjusted R^2^ of 0.36 for the regression model, see **Table 3**.

**Table 3:** Multivariable linear regression for quality of life in ANCA-associated vasculiits.

Independent variables	B (95% CI)	P-value	Beta
	**Age-adjusted Physical Component Summary**		
Knee force (% of pred.)	0.19 (0.08 to 0.29)	<0.001***	0.41
Baecke total index	1.6 (-0.1 to 3.3)	0.06	0.21
Age >69 years	13 (7 to 19)	<0.001***	0.52
Relapsing (vs new)	-9 (-14 to -3)	0.003**	-0.36
Elevated CRP (≥5mg/l)	-8 (-13 to -2)	0.005**	-0.32
Neurological vasculitis	-8 (-16 to 1)	0.07	-0.21
Female sex	-3 (-8 to 1)	0.16	-0.15
Prednisolone >5mg/d	0 (-5 to 5)	0.99	0.00
	**Age-adjusted Mental Component Summary**		
Baecke total index	2.4 (0.7 to 4.1)	0.006**	0.36
Prednisolone (>5mg/d)	-7.1 (-11.9 to -2.3)	0.005**	-0.38
ENT involvement	-6.9 (-11.7 to -2.1)	0.006**	-0.38
Age >69 years	3.9 (-2.0 to 9.8)	0.19	0.17
Relapsing (vs new)	2.2 (-3.4 to 7.9)	0.43	0.11
Female sex	-0.3 (-4.9 to 4.2)	0.88	-0.02

Multivariable final model of physical and mental quality of life. B coefficient of independent variable in linear regression. Beta standardised regression coefficient.

*P<0.05.

**P<0.01.

***P<0.001.

### Prednisolone use versus muscle strength and physical activity

Knee extension, hip flexion and elbow flexion forces were not correlated to current or cumulative prednisolone dose. Physical activity according to the accelerometer and Baecke total score showed significant negative correlations with cumulative prednisolone use, but not with current prednisolone dose, see **Table 4**. In paired measurements (n = 7), median elbow flexion, hip flexion and knee extension increased after discontinuation of GC therapy. However, this difference was only statistically significant for hip flexion force, see **S1 Fig**.

**Table 4:** Correlation of muscle strength and physical activity with prednisolone use.

Variable	Rho current prednisolone	P-value	Rho cumulative prednisolone	P-value
Muscle strength				
Knee extension (N)	-0.14	0.37	-0.03	0.86
% of predicted	-0.14	0.34	-0.13	0.38
Hip flexion (N)	-0.11	0.45	0.05	0.74
Elbow flexion (N)	0.01	0.97	0.18	0.23
% of predicted	0.04	0.81	0.17	0.26
Physical activity				
Actiwatch (kcount/d)	-0.23	0.25	-0.39	0.04*
Baecke total index	-0.17	0.24	-0.33	0.03*

Results of Spearman Rank correlation for measures of muscle strength and physical activity versus current prednisolone dose (mg/d) and cumulative use over the past six months (g).

*P<0.05

### Disease duration versus muscle strength and physical activity

In relapsing patients, a longer time after diagnosis showed a trend with lower knee extension force (% of predicted) (Rho = -0.32, P = 0.07) and lower Baecke index (Rho = -0.31, P = 0.08), but not with elbow flexion force (Rho = -0.23, P = 0.19). By contrast, in newly diagnosed patients, a longer time after diagnosis was associated with a higher Baecke index (Rho = 0.58, P = 0.04). Time after diagnosis still showed no significant correlation with knee extension force (Rho = -0.08, P = 0.79) or elbow flexion force (Rho = -0.17, P = 0.60) in newly diagnosed patients.

Patients with knee extension force below 100% of predicted had significantly longer median follow-up (118 months, IQR 30–201) compared to patients with at least 100% of predicted knee extension force (22 months, IQR 7–80), P = 0.006. This remained significant (P = 0.03) after correction for age, sex, Baecke total score and cumulative prednisolone use. Patients with elbow flexion force below 100% of predicted did not have a significantly longer follow-up (median 123, IQR 32–202 months if below 100%; median 36, IQR 11–118 months if at or above 100% of predicted; P = 0.13).

## Discussion

In this study, we found that the majority of AAV patients have a muscle strength below their predicted values based on age, sex, height and weight [18], and that physical QoL was significantly reduced in AAV patients compared to healthy norm values [27]. We also identified an association of self-reported physical activity measured by the Baecke questionnaire with mental QoL in AAV.

The reduced leg muscle strength in AAV patients, found in this and previous studies [12,29], might be the result of steroid myopathy. Indeed, studies in several other disease populations have previously shown associations between chronic glucocorticoid use and reduced muscle strength [30–32]. While leg muscle strength did not show a correlation with prednisolone use in the present cross-sectional study, this might be explained by large inter-individual variation in muscle strength and GC sensitivity. Inclusion of muscle imaging to assess for typical signs of muscle atrophy, such as ultrasonography or MRI, might be interesting for a future study.

Interestingly, relapsing patients with longer disease duration more frequently had a muscle strength below norm values. This suggests that accumulating damage from relapses of AAV and treatment of these relapses results in a reduction of muscle strength over time.

Physical activity, as measured using an accelerometer and the Baecke questionnaire, showed a negative association with cumulative prednisolone exposure, as well as a positive association with follow-up time in newly diagnosed patients. This indicates that prednisolone therapy negatively impacts exercise capacity and that physical activity increases when tapering prednisolone. Alternatively, the association might be confounded my more recent disease activity in patients with a higher cumulative prednisolone exposure in the past 6 months, as disease activity might also result in reduced physical activity. Contrary to our expectations, we did not find an association of muscle strength with cumulative prednisolone dose.

In agreement with earlier studies [4,5], especially the age-adjusted PCS was reduced in AAV patients compared to general population norms [27]. Leg muscle strength was independently associated with PCS. Therefore, muscle strength might be part of the explanation for reduced QoL in vasculitis patients. Leg muscle strength showed only a positive trend with physical activity. Also, leg muscle strength, in contrast to self-reported physical activity, was associated with PCS in multivariable linear regression. This suggest that muscle strength directly affects physical QoL, not (only) through physical activity. Physical activity was a main factor associated with mental QoL. Based on these results, interventions focusing on improving muscle strength and exercise capacity might improve both physical and mental QoL in AAV patients.

This study has several limitations. First, due to logistic reasons, only some patients received accelerometer and bioelectric impedance analysis (BIA) measurements, limiting the sample size for these measurements. For this reason, these measurements were not included in linear regression analyses. Furthermore, due to the cross-sectional nature of the study, inferences about causality cannot be made and statistical power is limited by inter-individual variation in variables measured. Also, no matched control group was included, requiring comparison to literature values from an unmatched general population. Lastly, generic questionnaires were used for the study. While this enables comparison with reference values from literature, the questionnaires are less sensitive to change than disease-specific questionnaires would be.

Our findings that most AAV patients have a lower muscle strength than expected based on healthy population norms and that muscle strength and self-reported physical activity are positively associated with QoL suggest that AAV patients might benefit from interventions aimed at improving muscle strength and physical activity. Studies in other disease populations have demonstrated clinically relevant improvements with simple interventions. For example, improvement of fatigue and physical function was achieved in Rheumatoid Arthritis patients by giving them a pedometer and a step-monitoring diary [33]. As fatigue negatively influences QoL in AAV [34], and self-reported physical activity was positively related to QoL in the present study, physical activity interventions might also reduce fatigue and improve QoL in AAV patients.

In conclusion, the majority of AAV patients have reduced leg muscle strength and physical QoL compared to norm values. Knee extension strength is independently associated with physical QoL, while self-reported physical activity is independently associated with mental QoL. Therefore, interventions promoting leg muscle force and physical activity might improve both aspects of QoL and should be evaluated in clinical trials.

## Supporting information

S1 Table Intra-rater reliability of handheld dynamometry muscle groups, overall and per assessor. Results shown are intraclass correlation coefficients (ICC) with 95% conficence intervals.
(DOCX)

S1 Fig Paired comparisons of muscle strength during and after high-dose prednisolone. Muscle strength of individual patients at visit V1 (prednisolone dose > = 30mg/d) and visit V2 (< = 2.5 mg/d) for elbow flexion (A), hip flexion (B) and knee extension (C). *P<0.05.
(DOCX)
