# Supplementary material for: Leg muscle strength is reduced and is associated with physical quality of life in Antineutrophil cytoplasmic antibody-associated vasculitis
Source: PLoS One. 2019 Feb 4;14(2):e0211895. doi: 10.1371/journal.pone.0211895 (PMC6361463; doi:10.1371/journal.pone.0211895)
Supplement: S1 Table — Results shown are intraclass correlation coefficients (ICC) with 95% conficence intervals. (DOCX) [file pone.0211895.s001.docx]

**S1 table. Intra-rater reliability of handheld dynamometry muscle groups, overall and per assessor.**

| **Muscle group** | **Overall (n=48)** | **Assessor A (n=23)** | **Assessor B (n=5)** | **Assessor C (n=6)** | **Assessor D (n=14)** |
| --- | --- | --- | --- | --- | --- |
| Elbow flexion (n=45) | 0.91 (0.85 to 0.95) | 0.91 (0.82 to 0.97) | 0.70 (0.27 to 0.96) | 0.93 (0.78 to 0.99) | 0.73 (0.48 to 0.90) |
| Hip flexion (n=46) | 0.86 (0.78 to 0.92) | 0.84 (0.70 to 0.93) | 0.81 (0.49 to 0.98) | 0.96 (0.86 to 0.99) | 0.76 (0.54 to 0.92) |
| Knee extension (n=46) | 0.83 (0.74 to 0.90) | 0.86 (0.73 to 0.95) | 0.81 (0.45 to 0.98) | 0.83 (0.51 o 0.98) | 0.66 (0.40 to 0.86) |

Results shown are intraclass correlation coefficients (ICC) with 95% conficence intervals.
