# Supplementary material for: Leg muscle strength is reduced and is associated with physical quality of life in Antineutrophil cytoplasmic antibody-associated vasculitis
Source: PLoS One. 2019 Feb 4;14(2):e0211895. doi: 10.1371/journal.pone.0211895 (PMC6361463; doi:10.1371/journal.pone.0211895)
Supplement: S1 Fig — Muscle strength of individual patients at visit V1 (prednisolone dose > = 30mg/d) and visit V2 (< = 2.5 mg/d) for elbow flexion (A), hip flexion (B) and knee extension (C). *P<0.05. (DOCX) [file pone.0211895.s002.docx]

**S1 Fig. Paired comparisons of muscle strength during and after high-dose prednisolone.**

**
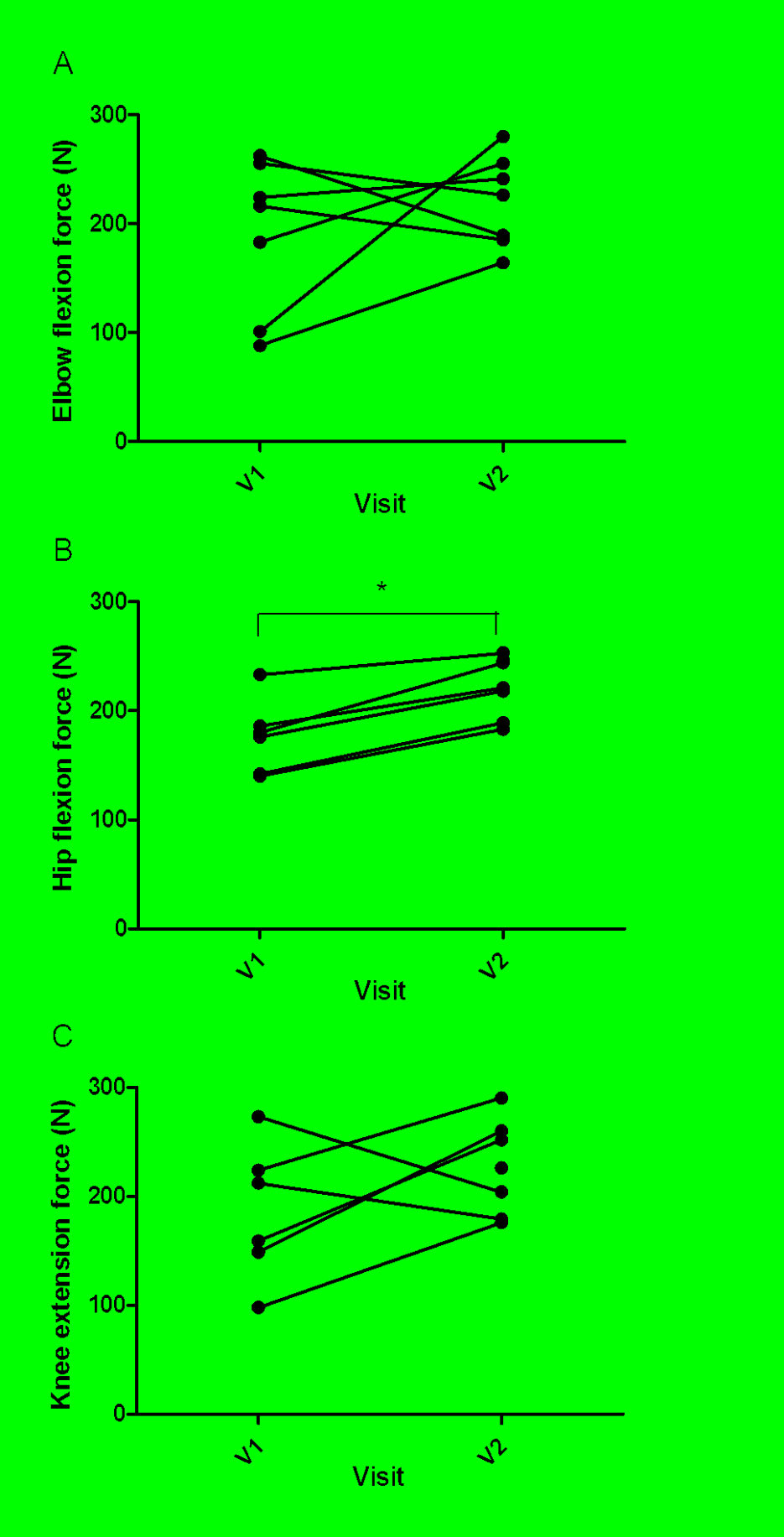
**

Muscle strength of individual patients at visit V1 (prednisolone dose >= 30mg/d) and visit V2 (<= 2.5 mg/d) for elbow flexion (A), hip flexion (B) and knee extension (C). *P<0.05.
